# Understanding and addressing COVID-19 vaccine hesitancy in low and middle income countries and in people with severe mental illness: Overview and recommendations for Latin America and the Caribbean

**DOI:** 10.3389/fpsyt.2022.910410

**Published:** 2022-09-13

**Authors:** Clara Gitahy Falcão Faria, Ursula Medeiros Araujo de Matos, Liana Llado-Medina, Victor Pereira-Sanchez, Rafael Freire, Antonio Egidio Nardi

**Affiliations:** ^1^Laboratory of Panic and Respiration, Federal University of Rio de Janeiro (UFRJ), Rio de Janeiro, Brazil; ^2^School of Medicine, University of Puerto Rico, Bayamón, Puerto Rico; ^3^Department of Psychiatry, Columbia University, New York, NY, United States; ^4^Division of Translational Epidemiology and Mental Health Equity, New York State Psychiatric Institute, New York, NY, United States; ^5^Department of Child and Adolescent Psychiatry, New York University Grossman School of Medicine, New York, NY, United States; ^6^Department of Psychiatry, Amoud University, Borama, Somalia; ^7^Department of Psychiatry and Center for Neuroscience Studies, Queen's University, Kingston, ON, Canada

**Keywords:** vaccine hesitancy, severe mental illness, mental health, COVID-19, Latin America and the Caribbean

## Abstract

Despite the speedy development of vaccines for COVID-19, their rollout has posed a major public health challenge, as vaccine hesitancy (VH) and refusal are high. Addressing vaccine hesitancy is a multifactorial and context-dependent challenge. This perspective focuses on VH in the world region of Latin America and the Caribbean (LAC) and includes people suffering from severe mental illness, therefore covering populations and subpopulations often neglected in scientific literature. We present an overview of VH in LAC countries, discussing its global and historical context. Vaccine uptake has shown to widely vary across different subregions of LAC. Current data points to a possible correlation between societal polarization and vaccination, especially in countries going through political crises such as Brazil, Colombia, and Venezuela. Poor accessibility remains an additional important factor decreasing vaccination rollout in LAC countries and even further, in the whole Global South. Regarding patients with severe mental illness in LAC, and worldwide, it is paramount to include them in priority groups for immunization and monitor their vaccination coverage through public health indicators.

## Introduction

Since the World Health Organization (WHO) declared the coronavirus pandemic (COVID-19) in March 2020, the outbreak has brought an unparalleled public health crisis. Initially, different preventive efforts varying in strictness and enforcement were adopted in response to it: physical distancing, lockdowns (1), canceling elective procedures (2), and redeployment of health care professionals (3). However, implementing and sustaining those measures to their full extent proved to be a significant challenge (1), and the development of a safe and affordable vaccine soon became a vital need. Scientific efforts were carried out with unprecedented speed and global coordination and investment, and by the end of 2020 there were 240 vaccines in development, with 9 in final stages of testing (4).

Despite the rapid development and approval of vaccines, their rollout has posed another significant challenge, as vaccine hesitancy (VH) and refusal are high in part of the general public (5), reaching as high as 40% in Russia and 26% in France as of January 2021 (6, 7). Besides vaccine availability, in order to reduce morbidity and mortality from COVID-19, a high vaccine uptake is necessary to reach herd immunity (8). Experience from previous pandemics and public health emergencies shows that addressing VH is a multifactorial and context-dependent challenge that must be addressed simultaneously at global, regional, and national levels (8–11).

Latin America and the Caribbean (LAC) is a world region home to a vast and young portion of the world population spread around many different low-and-middle income countries (LMIC) that largely share cultural roots, language, and religious beliefs in a unique way. Its demographic and cultural characteristics, the actual, devastating and unequal impact of the pandemic in LAC countries (12), and the traditional marginalization of this world region in scientific literature, dominated by Anglo-Saxon and high-income countries, provides a need and an opportunity to explore the state and challenges of vaccine rollout there, and a discussion of the unique cultural, religious, and mental health aspects associated to VH in this region. In this perspective paper, a group of mental health clinicians and researchers in the Americas present an overview of attitudes and hesitancy toward COVID-19 vaccines in general and in LAC countries, discussing its global and historical context. We will also address the cultural and religious factors contributing to the current scenario and particularly, as concerning people living with mental illness. Furthermore, we will outline potential strategies to improve vaccination intention (VI) and public trust in the LAC region.

## Vaccine hesitancy and anti-vaccine culture before and during COVID-19

VH is not a new phenomenon (13), rather it has been described since the inception of vaccines in the 1870s in England (13). In the USA, the anti-vaccination movement and a state government clashed in the landmark case *Jacobson v. Massachusetts*, which ruled that the state could mandate vaccination by law in order to protect the population (13). Similarly to the strategy being adopted by some countries today, in the nineteenth century many vaccination mandates were imposed in order to contain smallpox outbreaks (14). Those measures sparked resistance from liberal sectors of society which argued that they constituted a violation of civil liberties (15). Nowadays, anti-vaccination activism poses an even larger resistance as it harnesses powerful platforms able to reach big audiences worldwide, since the internet has become a major source of health information for the public (16, 17).

It is important to conceptualize the contemporary anti-vaccination movement as a spectrum of beliefs and concerns rather than a two-dimensional concept (18). Studies analyzing the vaccine hesitancy movement before COVID-19 show that VH is often demonstrated by postponing vaccines and increasing the delay between doses rather than complete refusal, which represents a smaller percentage of the anti-vaccination movement (18).

Culturally, before COVID-19 era, two main factors influenced VH: first, the fact that for a long time the diseases that vaccines prevented were largely unknown to the population (18, 19) thanks to the success of previous vaccination rollouts, therefore resulting in a low perceived benefit of vaccines when compared to the perceived risks. The trend that has been observed by public health specialists is that as an outbreak of a preventable disease occurs, vaccination rates improve (18). Secondly, highly publicized research that was later retracted led to a major public backlash against vaccines, as it falsely related the measles, mumps, and rubella vaccines to autism (20, 21). When it came to COVID-19, even before a vaccine was available, the terms “mercury” and “autism” were widely popular Google searches in association with the upcoming vaccine (22).

Furthermore, there is a group where VH data is particularly lacking: mental health patients. Recent studies (23–25) have shown that people experiencing severe mental health conditions are more likely to face longer hospitalizations due to COVID-19 and suffer worse outcomes and mortality rates, thus making it necessary to prioritize their vaccination and further explore VH in this group. Although VH has been widely explored in the general population and the development of the COVID-19 vaccine has been widely covered by the media, there have not been peer-reviewed studies exploring how the anti-vaccination movement affects people with severe mental illness, specifically those who experience persecutory delusions (24, 25). Recently, one study in the UK looked at the relationships of mental health diagnosis and symptoms of mental distress with VH in a general population sample and found no association (23). However, there is no data on community-based or in patient samples, which could potentially reveal a very different scenario.

### Strategies to increase vaccination uptake

One of the most defended strategies to increase vaccination coverage consists of increasing government's commitment and transparency toward health policies and promoting social economic growth for the society at large (26). This might be very effective in the long term, as it decreases mistrust from the population toward pharmaceutical and governmental organizations. However, the implementation of these measures would not address the problem promptly, and it requires profound structural changes in governmental and health systems all over the globe, particularly in underdeveloped nations, where poor health access and social inequalities are important barriers to vaccine uptake.

Among short-term strategies, those that tackle misinformation are of uttermost importance. Governments and health organizations should focus on conveying the message that vaccines are effective and safe while demonstrating the competence and reliability of the institutions that deliver them (27). However, recent research has shown that combating *fake news* and providing more information is not sufficient to change behavior (28). One of the factors that was shown to influence negatively on VH is a poor relationship with healthcare providers, pointing out to the importance of the rapport between patients and healthcare professionals (29). Moreover, in the case of COVID-19 vaccines, for the pharmaceutical industry and health and government authorities the emphasis should be to prove and convey that no developmental or regulatory corners were cut in the development and approval process, which were facilitated by extensive prior research, unprecedented levels of international collaboration among researchers, and massive public investment in research, development and manufacturing capacity.

Since the start of the pandemic, the mortality risk in patients who have suffered from COVID-19 has been studied. It has been found that, in individuals who were recipients of the Pfizer-BioNTech, Moderna, or Janssen vaccines there was a lower mortality risk that in unvaccinated comparison groups. After these comparison groups were analyzed and stratified by age, sex, race and ethnicity, they still showed a lower mortality in vaccinated adults compared to non-vaccinated adults. Therefore, the lower mortality risk after COVID-19 vaccination implies that there are beneficial vaccine effects on these individuals. Additionally, hospitalized individuals due to COVID-19 were less likely to have an mRNA COVID-19 vaccine (30). Spreading evidence based information is one potential way to increase vaccination uptake (31).

## Vaccine hesitancy and anti-vaccine culture in Latin America and the Caribbean

In LAC countries vaccine uptake is heterogeneous and immunization rates are prone to vary across different subregions (32–37). The extent to which people believe in COVID-19 conspiracy theories varies significantly across LAC countries as well as by socio-demographic characteristics (36). For instance, VI in Trinidad and Tobago was estimated at 62.8%, which largely contrasts with other countries such as Cuba, Chile, Uruguay, Argentina, and Brazil, which have the highest number of administered doses per 100 habitants (34).

Some authors have suggested that a country's resilience to misinformation and conspiracy theories depends on several political and economic indicators such as the level of societal polarization, the amount of populist and partisan communication, the strength of public service media, and the adoption of social media (38–41). When it comes to LAC, many countries indeed confront higher societal polarization, besides from having weaker public service media systems compared to other Western nations. Many countries in LAC such as Venezuela, Brazil, Argentina, and Bolivia are undergoing intense economic and political crises, which are driving social division. Even though more research is warranted, current data points to a possible negative correlation between societal polarization and vaccination uptake in those nations. For instance, in Brazil, Ebeling et al. found that anti/pro COVID-19 vaccination stances are biased by political polarization, right and left, respectively (42).

In terms of other social, demographic, and cultural aspects, research indicates several risk factors to VH, with heterogeneity across studies. For instance, religion, healthcare access barriers, being part of a more conservative political party, and low education levels have been shown to have a positive correlation with VH (33–37). Age and gender also presented with divergent results across different studies: Urrunaga-Pastor et al. (9) found increased age to be protective, whilst other studies found it to be a risk factor. De Coninck et al. (38) correlated increased age with less misinformation and conspiracy beliefs, while Puri et al. (26) found older age as one of the risk factors for vulnerability for social media appeals. Regarding gender, some studies have put male gender as a risk factor for VH, however findings are contradictory, with other studies pointing to female gender as a risk factor (9).

## Discussion

Overall, VH has been shown to be influenced by (1) lack of confidence, which is the lack of trust in the vaccine or provider, (2) complacency, which is the perception that there is no value or a need for a vaccine, and (3) lack of convenience, which refers to the perceived lack of access or services toward vaccination. COVID-19 VH can be explained by a combination of both underlying issues common to VH in general, as well as to the public's particular concerns specific to the SARS-CoV-2 vaccines (34, 35). Mistrust and misinformation are among the main reasons for missed vaccinations. Many individuals, mainly those who have been historically marginalized in their home countries, may find it difficult to trust their government, health system, and the pharmaceutical industry. Limited access to evidence-based information combined with social media spread of inaccurate yet appealing narratives can further propagate misinformation and fear, thus ramping up immunization refusal (32).

On the other hand, fear appraisal strategies have been used for health promotion for many years. It is understood that even though fear can change current behavior, it cannot produce a long lasting and real change. For instance, people can get vaccinated for COVID-19 due to extensive fear-promoting propaganda or governmental pressure, but they will not be truly aware of the importance of vaccination (38, 39). Currently, refusal occurs in many cases due to fear toward vaccines'—mainly COVID RNA vaccines—safety. As opposed to fear-promotion, empathetic information delivery strategies that address public's fears could be more effective and sustainable in promoting long lasting change.

Moreover, besides mistrust and misinformation, campaigns hampered up by the anti-vaccination movement, which are commonplace worldwide, healthcare and vaccine access are huge challenges in LAC, as some countries as Venezuela, for example, are facing severe political crises (33, 43). Others, such as Peru, face deep inequalities with some sectors of society receiving the vaccine before the rest of the population based on socioeconomic advantage rather than medical priority (44). However, some countries, such as Brazil, had a mostly successful vaccination rollout, maintaining a low VH rate: in a survey conducted with 173,000 participants VH was around 10.5% (45). Brazil is an interesting case study as, before the COVID-19 pandemic, the country already had an established successful national public immunization program rolling for 46 years, despite its many inequalities (46). It has been suggested that, despite a conservative government in power repeatedly accused of spreading misinformation about vaccines, VH in Brazil is still low due to the country's longstanding history of immunization thanks to the massive investment over decades in the Brazilian National Immunization Program. In summary, the VH landscape in LAC is highly heterogeneous and still understudied (34, 35, 37).

In addition, there is a group needing urgent attention not only in LAC countries but also worldwide when it comes to vaccination intention and hesitancy: individuals with severe mental illness. This is a particularly big challenge in the LAC context, as many countries such as Brazil, Colombia, Costa Rica, and Peru are facing the challenge to provide treatment for a growing number of patients while transitioning to a community-based care model, and face shortage of mental health services provision in the community (47, 48). It is also important to highlight that many people with severe mental illness in LAC are socially marginalized and face many difficulties in terms of access to health and social services. There seems to be no research looking into the factors that influence vaccination uptake in patients living with a mental illness, however, an important factor in the general population is the rapport with the main healthcare provider, as previously discussed (31). This research gap is even more appalling since people with severe mental health disorders are at higher risk of being infected by SARS-CoV-2 and have increased COVID-19 associated mortality rates, as also previously mentioned (24–26). Especially for a population group facing difficulties to access healthcare in general, we suggest that mental health practitioners should play a pivotal role in the understanding and addressing of their VH and uptake.

### Strategies to increase vaccination coverage in LAC, especially in patients with severe mental illness

Besides delivering transparent, trustworthy, and empathetic information to the public and encouraging active demand for vaccination, facilitating health service access can contribute to the number of immunizations of those individuals that are not opposed to the vaccine, but also will not actively seek immunization, following the public health principle of “making healthy choices, easy choices” (49). Poor accessibility remains an important factor that decreases vaccination coverage in LAC countries and even further, in the whole Global South (50). Regarding patients with severe mental illness in Latin America and the Caribbean and worldwide, it is necessary to transform them into priority groups for immunization and monitor their vaccination coverage through public health indicators. One possible strategy is to partner with community mental health centers and inpatient units, making vaccination more accessible to those marginalized groups.

## Conclusion

Our perspective identified a major research gap in terms of VH in LAC populations, including people with severe mental illness. Further work is needed to develop region and country-based strategies to increase immunization rates tailored to common and unique socio-cultural factors. Regarding LAC, the VH landscape is highly heterogeneous. Among the factors that are believed to contribute to a decreased vaccination coverage in LAC countries, we can name unequal access, mainly in rural areas; political and socio-economic crisis; societal polarization; and misinformation propagated by social media paired with poor access to evidence-based health information.

To summarize, the COVID-19 vaccination process is one of the greatest global health challenges of our time. In order to achieve the global health agenda of increased vaccination coverage, no marginalized group should be left behind. We would like to strongly emphasize the need for further investment in research in VH and associated factors in LMIC populations, including all of LAC, and especially including people with severe mental health illness.

## Data availability statement

The original contributions presented in the study are included in the article/supplementary material, further inquiries can be directed to the corresponding author/s.

## Ethics statement

Ethical review and approval was not required for the study on human participants in accordance with the local legislation and institutional requirements. Written informed consent for participation was not required for this study in accordance with the national legislation and the institutional requirements.

## Author contributions

CF, UM, and LL-M created the concept of the manuscript, conducted the literature review, and wrote it. VP-S helped create the concept of the manuscript, supervised all steps of the work, and reviewed the manuscript draft. RF and AN provided guidance and supervision, and proofread the manuscript. All authors approved the final version of the manuscript and assumed their responsibility as coauthors.

## Funding

Funds from Cnpq (Brazilian National Council for Scientific Development) have been granted to cover article processing fees for open access publication of this work.

## Conflict of interest

The authors declare that the research was conducted in the absence of any commercial or financial relationships that could be construed as a potential conflict of interest. The Handling Editor SE declared a past co-authorship/collaboration with the Author VP-S.

## Publisher's note

All claims expressed in this article are solely those of the authors and do not necessarily represent those of their affiliated organizations, or those of the publisher, the editors and the reviewers. Any product that may be evaluated in this article, or claim that may be made by its manufacturer, is not guaranteed or endorsed by the publisher.
